# Djaffa Mountains guereza (*Colobus guereza gallarum*) abundance in forests of the Ahmar Mountains, Ethiopia

**DOI:** 10.5194/pb-10-13-2023

**Published:** 2023-10-20

**Authors:** Chala Adugna Kufa, Afework Bekele, Anagaw Atickem, Dietmar Zinner

**Affiliations:** 1 Department of Zoological Sciences, College of Natural Sciences, Addis Ababa University, P.O. Box 1176, Addis Ababa, Ethiopia; 2 Department of Biology, Faculty of Natural and Computational Sciences, Woldia University, P.O. Box 400, Woldia, Ethiopia; 3 Cognitive Ethology Laboratory, German Primate Center (DPZ), Leibniz Institute for Primate Research, Kellnerweg 4, 37077 Göttingen, Germany; 4 Department of Primate Cognition, Georg August University, 37083 Göttingen, Germany; 5 Leibniz ScienceCampus “Primate Cognition”, 37077 Göttingen, Germany

## Abstract

The size and density of a population are essential parameters in primate ecology and conservation. Such information, however, is still scarce for many forest primate species. The Djaffa Mountains guereza (*Colobus guereza gallarum*) is an endemic Ethiopian taxon for which data about its distribution and population size are missing. Therefore, we aimed to estimate the abundance and population size of the Djaffa Mountains guereza in four forests in the Ahmar Mountains southeast of the Ethiopian Rift Valley. We conducted line-transect surveys in the forests. Within an area of 183 km ${}^{2}$ , we sampled 19 transects covering a distance of 75.9 km. We encountered 73 guereza clusters which most likely represent social groups. Since the detection distances and cluster sizes did not differ among the four forests, we applied a conventional distance sampling (CDS) model and estimated a population density of 20.6 clusters per square kilometer, i.e., 109.6 individuals per square kilometer or 20 061 individuals within the complete study area. This abundance is relatively high compared to other *C. guereza* taxa. However, given that the habitat and population of C . g . *gallarum* are already highly fragmented, further monitoring of the population and exploration of the possibilities of reconnecting its habitat should be priorities for the conservation of this taxon.

chalaadugna@gmail.com]Chala AdugnaKufa

## 1 Introduction

Large parts of the global biodiversity are threatened by extinction, including many primate species. Most primate species (93 % of the species) are experiencing population declines (Estrada and Garber, 2022), and the International Union for Conservation of Nature (IUCN) lists over 65 % of primate species as “Vulnerable”, “Endangered”, or “Critically Endangered” (Fernández et al., 2022). The reasons for this negative trend are generally well-known and include the destruction, fragmentation, and conversion of primate habitats; hunting; and illegal trade (Estrada et al., 2017). Future human population growth, agricultural expansion, and climate change are expected to accelerate the decline of primate populations (Estrada et al., 2020; Estrada and Garber, 2022; Pinto et al., 2023). To develop species-specific conservation strategies and/or to monitor implemented conservation measures, estimating densities and abundances of populations is essential (Jachmann, 2001; Marques, 2001; Keeping and Pelletier, 2014; Kiffner et al., 2022a). This is particularly important for threatened species living in already human-modified landscapes (Cavada et al., 2016). Animal population surveys are therefore an essential contribution to the successful conservation of species (Ogutu et al., 2006; Santini et al., 2022).

**Figure 1:**
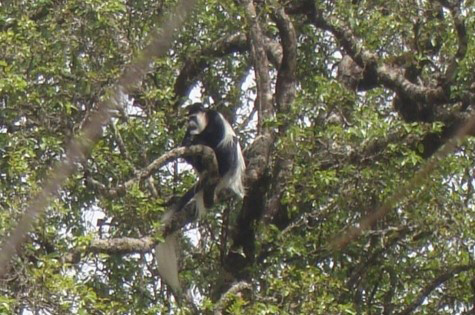
Male Djaffa Mountains guereza (*Colobus guereza gallarum*) in Hades Forest, 2021 (photo: Chala A. Kufa).

In recent decades, Ethiopia has experienced a severe loss of forest habitats in almost all regions of the country and thus the primary habitats for forest-dependent species, including several primate taxa (Yalden et al., 1977). One primate taxon that is most likely at extinction risk is the Djaffa Mountains guereza (*Colobus guereza gallarum* Neumann, 1902, hereinafter referred to as DMG; Fig. 1) because, in its supposed range, only a few forested areas remain (Kufa et al., 2022). DMG is a black-and-white colobus endemic to the Arsi and Ahmar Mountains in Ethiopia (Groves, 2001; Fashing and Oates, 2013, 2019; Zinner et al., 2019; Butynski and De Jong, 2022). However, its exact distribution is uncertain. In particular, information on the range boundaries between DMG and C . g . *guereza* is missing. The occurrence of DMGs west of the Arsi and Ahmar Mountains, e.g., in Munessa Forest, Wondo Genet Forest, Dale Forest, and Bale Mountains National Park, is questionable (Petros et al., 2018a, b, c; Menbere and Mekonen, 2019; Mekonen and Hailemariam, 2021). At the very least, these western populations do not carry DMG-specific mitochondrial haplotypes (Zinner et al., 2019; Tesfaye et al., 2021). Given the dramatic loss and degradation of forests in its range as well as its likely limited distribution, the DMG is most likely facing a severe risk of extinction (Kufa et al., 2022). However, due to poor knowledge of its population size and distribution, the DMG is listed as “Data Deficient” by the IUCN (Fashing and Oates, 2019).

In this study, we estimated the population density and size of DMGs in four of the remaining forests in the Ahmar Mountains of Ethiopia. We used line-transect distance sampling, which has been widely used to estimate the densities of diurnal arboreal primates in tropical forests (González-Solís et al., 2001; Buckland et al., 2010; Höing et al., 2013; Leca et al., 2013; Araldi et al., 2014; Omifolaji et al., 2020; Kiffner et al., 2022a, b). This method is relatively simple, rapid, and cost-effective (Buckland et al., 2001; Thomas et al., 2010). Furthermore, it is also used for sparsely distributed populations for which sampling needs to be efficient, populations that occur in well-defined clusters and at low or medium cluster density, and populations that are detected through a flushing response (Buckland et al., 2001).

## 2 Methods

### 2.1 Ethical statement

This research adhered to the legal requirements of Ethiopia, and there was no animal handling. Permission to carry out the study was obtained from the Department of Zoological Sciences, Addis Ababa University, and the Oromia Forest and Wildlife Enterprise.

### 2.2 Study sites

**Figure 2:**
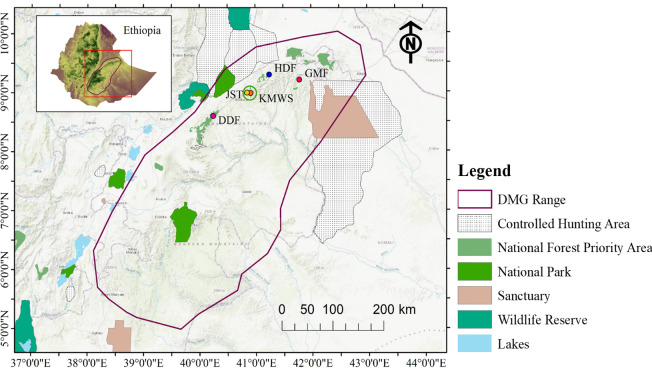
Geographic position of the study area in the Ahmar Mountains, Ethiopia, and locations of the four forest sites where we did the line-transect surveys on Djaffa Mountains guereza (DMG): Dindin Forest (DDF), Jallo Kuni-Muktar Wildlife Sanctuary (JKF, comprising two sites nearby: Jallo Sorroro Torgam – JST – and Kuni-Muktar Wildlife Sanctuary – KMWS), Hades Forest (HDF), and Gara Muleta Forest (GMF) (source of protected area shapefiles: UNEP-WCMC and IUCN, 2023).

We conducted our study in the Ahmar Mountains, a mountain range of the Ethiopian Highlands south of the Rift Valley in the Oromia regional state of Ethiopia (Fig. 2). The topography of the Ahmar Mountains is characterized by plateaus, rugged dissected mountains, deep valleys, gorges, and plains (Abdala et al., 2017). The climate of the study area receives a bimodal rainfall distribution (Fig. S1a–d), with a short rainy season between February and May, long rains between July and September, and a long dry period between October and January.

Originally, dry evergreen Afromontane forest dominated the region (Bishaw, 2001; Friis et al., 2010; Asefa et al., 2020). However, most parts of the Ahmar Mountains are now covered by wooded grasslands, with stands of exotic tree species such as *Eucalyptus* and bushland (Friis et al., 2010). The remaining forests in the region are often fragmented and degraded due to unsustainable use and persistent drought (Abdala et al., 2017).

**Table 1:** Characteristics of each study site in the Ahmar Mountains, Ethiopia: geographic coordinates in decimal degrees; elevation (m a.s.l.); size (km ${}^{2}$ ); mean ( ±SD ) annual precipitation (mm); mean ( ±SD ) annual temperature ( ${}^{\circ }$ C).

Name of site	Lat	Long	Elevation	Size	Vegetation	Precipitation	Temperature	Legal status ${}^{*}$	Status
Dindin Forest (DDF)	8.61581	40.26009	1980–3070	83	LCF	957 ± 130	21.4 ± 0.37	NFPA, CHA	Proposed
Jallo-Sorroro Torgam (JST)	9.01547	40.85807	1945–3025	48.3	SCF	836 ± 116	23.3 ± 0.37	NFPA	Proposed
Kuni-Muktar	8.98885	40.92519	2165–2950	16.8	SCF	959 ± 147	21.8 ± 0.36	CHA, WS	Designated (2000)
Wildlife Sanctuary (KMWS)									
Hades Forest (HDF)	9.31768	41.24319	2050–2750	6	LSF	994 ± 144	20.2 ± 0.36	NFPA	Proposed
Gara Muleta Forest (GMF)	9.25486	41.75294	2450–3370	29	SCF	1007 ± 154	18.8 ± 0.36	NFPA	Proposed

NFPA: national forest priority area; CHA: controlled hunting area; WS: wildlife sanctuary; LSF: large forest fragment (1–10 km ${}^{2})$ ; SCF: small continuous forest (11–50 km ${}^{2})$ ; LCF: large continuous forest ( > 50 km ${}^{2})$ . ${}^{*}$ Source: UNEP-WCMC and IUCN (2023).

**Figure 3:**
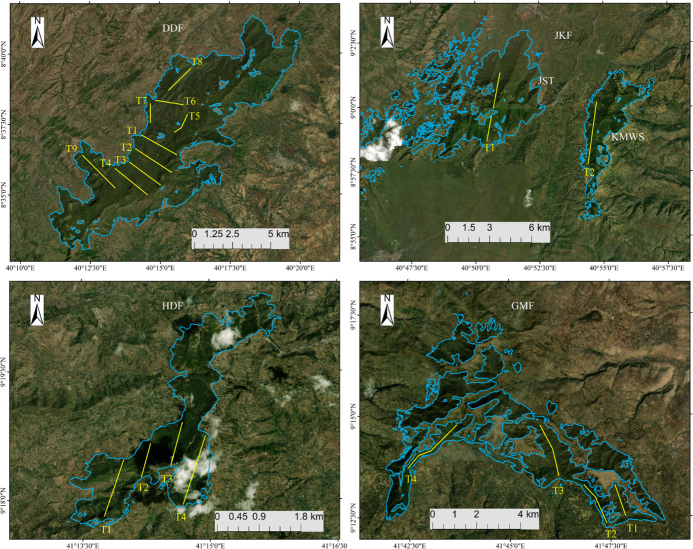
Positions of transects (yellow lines) within the four forests used for DMG surveys from 2020 to 2021: DDF, JKF, HDF, and GMF. The maps show the extents of the study sites for each forested area. Base map: ©Google Earth.

For our survey, we selected four forests (Table 1; Fig. 3) where DMGs had been reported in previous studies (Zinner et al., 2019; Kufa et al., 2022) and where we confirmed their presence in a pilot study (unpublished data). These forests have been managed at the regional level by the Oromia Forest and Wildlife Enterprise (OFWE), which is advocating the preservation and protection of the natural forest through a participatory forest management approach. As a restoration practice, *Juniperus procera*, *Cupressus lusitanica*, *Hagenia abyssinica*, and *Croton macrostachyus* have been planted by the OFWE in collaboration with the local community.

The four forests differ slightly in their ecologies (Table 1). DDF is one of the remnants of the natural forests, marking a large continuous forest in the eastern part of the country. The dominant woody vegetation found in the forest includes *Juniperus procera*, *Olinia rochetiana*, *Maytenus addat*, *Maytenus undata*, *Podocarpus falcatus*, *Myrsine africana*, *Olea europaea, Maesa lanceolata*, *Myrsine melanophloeos,* and *Schefflera volkensii* (Shibru and Balcha, 2004).

JKF is a concession-controlled hunting area that comprises two fragmented forests, JST and KMWS. The concession area is represented by woody tree species such as M . *lanceolata, J*.* procera, C*.* macrostachyus, Rhus glutinosa,* and *Acacia abyssinicus* (Reshad et al., 2020).

HDF consists mainly of dry Afromontane forest comprising 40–48 woody species (Teketay, 1997; Atomsa and Dibbisa, 2019). The dominant tree species are *C. macrostachyus*, *H. abyssinica*, *Schefflera abyssinica*, and *Prunus africana*.

GMF harbors diverse flora, with about 361 species of vascular plants, of which 45 (13 %) are endemic to Ethiopia (Teketay, 1996). The southern side of Gara Muleta is covered by a multi-story mixed deciduous forest dominated by *Ekebergia capensis* and *P. africana* associated with intermediate and lower-story species that include *Bersama abyssinica*, C . *macrostachyus*, *Dioscorea schimperiana*, *Erythrina brucei*, H .* abyssinica*, *Nuxia congesta*, O .* africana*, and S .* abyssinica* (Teketay, 1996).

### 2.3 Survey method, design, and data collection

As a method of estimating abundance, we conducted line-transect surveys in four forest sites in the range of DMG in the Ahmar Mountains, employing a conventional distance sampling (CDS) approach (Buckland et al., 2001). This approach only considers the perpendicular distance to estimate the detection probability, thus in turn inferring the population size and estimating the density of a detected animal (Buckland et al., 1993, 2001).

We generated 19 transect lines (Fig. 3; range: 1.8–7.0 km) on topological maps of the four forest fragments using ArcGIS 10.4.1 in a random sampling design (Thomas et al., 2010; Buckland et al., 2015). Of the 19 transects, 9 were in DDF, 2 in Jallo-Kuni Muktar Wildlife Sanctuary, 4 in Hades Forest, and 4 in Gara Muleta Forest, covering a total distance of 75.9 km (Table 2). We positioned the transects in parallel wherever possible, with 1 km between adjacent transects, except for transects 3 and 4 of GMF, where the distance is approximately 5 km.

**Table 2:** Number of transects per study site, lengths of transects, encounter rates, and mean cluster sizes per study site.

Site ID	Name of study site	Number of	Transect	Number of	Encounters per	Mean cluster
		transects	length (km)	encounters	kilometer	size ( ±SD )
DDF	Dindin Forest	9	39.3	41	1.04	5.5 ± 2.8
JST	Jallo-Sorroro Torgam	1	7	3	0.43	
KMWS	Kuni-Muktar Wildlife Sanctuary	1	5.2	4	0.78	
Combined	JKF	2	12.2	7	0.57	4.3 ± 2.0
HDF	Hades Forest	4	12.0	15	1.25	4.5 ± 1.9
GMF	Gara Muleta Forest	4	12.5	10	0.80	6.5 ± 1.7
All		19	75.9	73	0.96	5.3 ± 2.5

We did the fieldwork from December 2020 to September 2021. From 07:00 to 18:00 Eastern African Time, a team of three well-trained persons led by CAK walked slowly along each transect (1 km h ${}^{-1}$ ) (Peres, 1999; Plumptre et al., 2013). We assumed that our target species had daily travel distances similar to a closely related taxon, *C. g. occidentalis* (62 to 1360 m; Fashing, 2001a). We surveyed each transect once per season.

Colobus monkeys are relatively easy to detect because their movements, loud calls, and pelage make them conspicuous (Fig. 1). They often indicate their presence with loud calls that may be heard for more than 1 km. For each encounter, we recorded the (1) transect ID, (2) date and time of the sighting, (3) weather conditions, (4) sighting location along the transect using the Global Positioning System (Garmin^®^ GPSmap 76CSx), (5) number of individuals (cluster size), (6) perpendicular distance (visually estimated), and (7) initial detection cue (auditory or visual). The personnel estimated the perpendicular distance after they were carefully trained in how to estimate distances and until the errors in distance estimation were reduced to less than 2 m. We visually estimated the perpendicular distances to the center of each cluster at their first detection (Kiffner et al., 2022a).

The home range size of C .* guereza* ranges from 0.075 to 1 km ${}^{2}$ (Fashing, 2011), and daily travel distances vary from 390 to 600 m (Fashing, 2011). Core areas are usually defended, and groups keep a certain distance from each other (von Hippel, 1996). We therefore defined individuals > 50 m apart as members of different clusters (Teelen, 2007; Kiffner et al., 2022b).

### 2.4 Data analyses

We conducted our survey in four forests and two seasons, and since forest ecology and season (more or less good visibility) can affect detection distance (perpendicular distance), we first tested whether forest ID or season (dry and wet) affected the perpendicular distances. Second, we compared cluster sizes among the four forests and tested whether cluster size affected the perpendicular distances since larger groups might be easier to detect (shorter perpendicular distances). We did these tests in R 3.4.1 (R Core Team, 2022) and found no effects of forest ID, season, or cluster size on detection distances (see the Appendix; Fig. S2a–e). We therefore combined all our encounters in a single analysis.

Since we recorded the perpendicular distances to the nearest meter, we subsequently ordered them into four distance classes to better fit the detection function: 0–10 m, > 10–20 m, > 20–30 m, and > 30–50 m. We had only two encounters with perpendicular distances larger than 50 m (one with 75 m and a cluster size of 5 and one with 100 m and a cluster size of 2). We therefore truncated the distribution at 50 m to increase the robustness of the detection function (Buckland et al., 1993, 2001). To establish the detection models on a large enough sample size since 60–80 detections are advised for consistently producing accurate density estimations (Peres, 1999; Buckland et al., 2001) and our sample sizes do not support separate fitting of the detection function to the data in each stratum of the forest, we combined all data across the study sites for fitting a detection function.

Using the pooled encounters across the forests, we fitted four candidate detection models to check the performance of these functions (uniform, half-normal, hazard rate, and negative exponential) in the conventional distance sampling (CDS) in Distance 7.3 Release 2 (Thomas et al., 2010) (Table 3; Fig. S3a–d). This determines the basic model shape. We considered three series expansions or adjustment terms, i.e., cosine, Hermite polynomial, and simple polynomial (Table 3), to make the models more robust.

**Table 3:** Results from fitting different detection models for DMG across the four forest sites during the survey periods. These models are arranged by differences in Akaike's information criterion ( Δ AIC*) between each candidate model and the model with the lowest AIC value. The key functions are uniform (UN), half-normal (HN), hazard rate (HR), and negative exponential (NE). The adjustment terms are cosine (CS), simple polynomial (SP), and Hermite polynomial (HP). The number of parameters (“np”) is shown for each model. “Pa” is the estimated detection probability; “ESW” is the estimated strip width in meters, “ D ” is the population density, and “CV” is the coefficient of variation. Numbers in parentheses denote the 95 % confidence intervals.

Model (series)	np	Δ AIC*	AIC	Pa	ESW (m, 95 % CI)	D (95 % CI)	D (CV)
CDS: HN	1	0.00	186.75	0.47 (0.39–0.56)	23.31 (19.28–0.56)	109.62 (75.99–158.13)	0.183
CDS: UN (CS)	1	0.70	187.45	0.51 (0.45–0.57)	25.28 (22.49–28.42)	101.06 (72.05–141.76)	0.167
CDS: NE	1	0.88	187.64	0.33 (0.25–0.44)	16.60 (12.41–22.20)	153.96 (100.85–235.05)	0.214
CDS: HR	2	2.04	188.79	0.48 (0.36–0.63)	23.86 (18.05–31.54)	107.11 (70.69–162.28)	0.210

We selected the best model using the Akaike information criterion (AIC) (Akaike, 1973) and retained the half-normal key function with no adjustment term ( Δ AIC = 0.0; AIC = 186.75; Table 3). In addition, we considered the chi-squared goodness-of-fit test and the visual fit of the models as additional model selection criteria (Buckland et al., 2001). We used the chi-squared test to compare the number of observations in a given distance interval to the number expected under the fitted detection function (Table S1). Then, we computed the probability of detecting a cluster given it is in the covered area, which is used to correct the density estimation (i.e., the number of groups per square kilometer) across the study periods and to determine the effective strip width (ESW, which is the distance at which as many objects are seen) (Thomas et al., 2010).

We computed the mean size of clusters in the population, the density of clusters, the population density, and the abundance of clusters for all the data combined. Since predicted cluster size estimation based on the size-biased regression method indicated warnings, we used the mean of the observed clusters as the basis for expected cluster size estimation. We also computed relative abundance (i.e., an index of abundance, usually presented as an encounter rate (ER) of objects recorded per unit of distance; Campbell et al., 2016; Fewster et al., 2009). We finally estimated the population size of the study taxon by multiplying the global lumped population density by the total area of the studied forests (183 km ${}^{2})$ . We quantified parameter estimates of uncertainty or variance using the standard error (SE), percent of the coefficient of variation (%CV), and 95 % confidence intervals (CIs) in the analytic variance estimation method in the distance.

## 3 Results

### 3.1 Encounter rate and cluster size

In total, we encountered DMGs 73 times (Table 2), with the highest encounter rate in HDF (1.25 per kilometer), followed by DDF (1.04 per kilometer), GMF (0.80 per kilometer), and JKF (0.57 per kilometer). The overall encounter rate was 0.96 per kilometer. The number of DMG individuals per cluster varied from 1 to 15 ( N=73 ), with an overall mean cluster size of 5.3±2.5 ( mean±SD ).

### 3.2 Modeling the detection function

**Figure 4:**
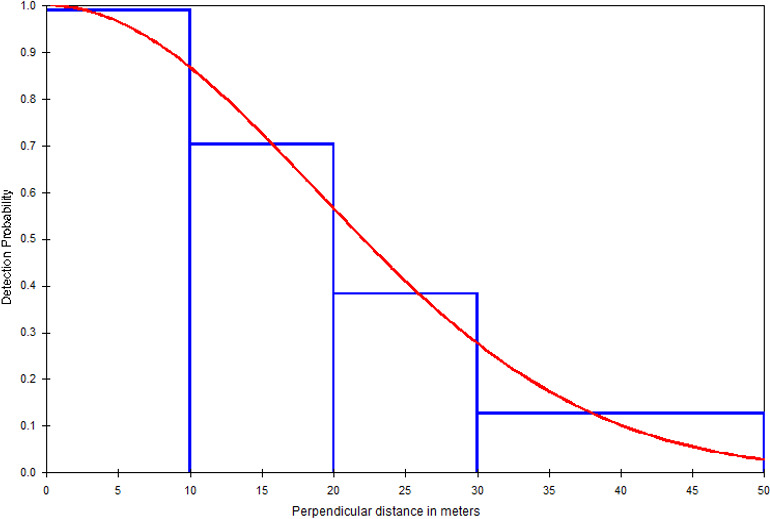
Frequency histogram of observed perpendicular distances truncated at 50 m and the detection function (red line) *for DMG* (half-normal key function).

The result of the model selection suggested a model with a half-normal key function and no simple polynomial adjustment as the best detection model (Table 3). The chi-squared goodness-of-fit tests show that a detection function model provides an adequate fit to the grouped distance data ( χ2=0.1682 , df = 2, P=0.9193 ; Table S1). Figure 4 depicts the fitted detection function averaged over the observed perpendicular distance for the half-normal model. Histograms of detected distances show higher detections close to the line transect, fitting the assumption of distance sampling analyses (Fig. 4).

### 3.3 Population size and density estimate of Djaffa Mountains guerezas

We estimated the group density as 20.6 clusters per square kilometer (95 % CI: 14.5–29.3; %CV = 17.5; df = 35.1) and the population density of DMGs as 109.6 individuals per square kilometer (95 % CI: 76.0–158.1; %CV = 18.3 %; df = 42.3) (Table 4). The estimated total population size for the complete study area of 183 km ${}^{2}$ was 20 061 individuals (95 % CI: 13 907–28 938; %CV = 18.3).

**Table 4:** Parameter estimate analysis and inference for DMGs across the forest fragments during the survey periods using the best model selected, i.e., half-normal key $k(y)=\text{Exp}(-y^{**}2/(2^{*}A(1{)}^{**}2)$ . “ A(1) ” is the i th parameter in the estimated probability density function (pdf); “ f (0)” is $1/u(W^{*}p)$ , the effective detection area for line transects, which is the value of the pdf at zero for line transects; “ p ” is the estimated detection probability; “ESW” is the effective strip width for line transects $W^{*}p$ ; “n/L” is the encounter rates; “DS” is the estimate of the density of clusters; “ E (S)” is the estimate of the expected value of a cluster size; “ D ” is the estimate of the density of animals; and “ N ” is the estimate of the number of animals in a specified area.

Parameter	Point estimate	SE	%CV	df	95 % CI
A (1)	18.74	1.91	–	–	–
f (0)	0.04	0.004	9.55	72.00	0.04–0.05
p	0.47	0.05	9.55	72.00	0.39–0.56
ESW	23.31	2.23	9.55	72.00	19.28–28.19
n/L	0.96	–	14.60	18.00	0.71–1.31
DS	20.63	3.60	17.45	35.07	14.51–29.31
E (S)	5.32	0.30	5.55	72.00	4.76–5.94
D	109.62	20.07	18.31	42.32	75.99–158.13
N	20 061	3673.3	18.31	42.32	13 907–28 938

## 4 Discussion

During our field surveys, we encountered 73 clusters of DMGs. The estimated population parameters of DMGs were 20.6 clusters per square kilometer, 109.6 individuals per square kilometer, or 20 061 individuals for the complete study area of 183 km ${}^{2}$ . The mean cluster size of DMGs (5.3 individuals) did not differ among the four study sites. If one assumes that the cluster size corresponds to the size of their social groups, this figure is at the lower end of reported average group sizes for other *C. guereza* subspecies (5.4 to 19; Fashing, 2011). It remains an open question whether the relatively low group size of DMG is taxon-specific or is caused by any ecological factor.

Our encounter rate (0.96 clusters per kilometer) is comparable to other colobus sites (1.2 clusters per kilometer, Kakamega Forest, Kenya, Fashing and Cords, 2000; 1.65 clusters per kilometer, central Ethiopia, Yazezew et al., 2022). Similarly, our population density estimate (109.6 individuals per square kilometer) fits into the range of population densities found at other sites (4.9 to 150 individuals per square kilometer, with one outlier of 315 individuals per square kilometer; Table 5), although one has to be careful when directly comparing population densities from different sites and with different ecologies, and most importantly when different census methods have been applied (Spaan et al., 2019; Kiffner et al., 2022b). Since there is a lack of baseline data, we are also not able to establish with certainty whether the population of DMG has declined, increased, or remained unaffected due to changes that occurred in the fragmented forests of the Ahmar Mountains.

**Table 5:** Population parameters of the African black-and-white colobus.

Taxon	Site	Area	Cluster density	Individual density	Reference
		(km ${}^{2}$ )	(clusters/km ${}^{2}$ )	(individuals/km ${}^{2}$ )	
*C. g. gallarum*	Ahmar Mts, ETH	183	20.6	109.6	This study
*C. g. guereza*	Wof-Washa Forest, ETH	25.6	14.3	94.4	Yazezew et al. (2022)
	Borena-Sayint NP, ETH	19	14.8	114.2	Ibrahim et al. (2017)
	Bole Valley, ETH	0.1	–	315	Dunbar (1987)
*C. g. occidentalis*	Kakamega, KEN	–	–	150	Fashing (2001a, b)
	Kibale NP, UGA	766	0.8–9.1	26	Chapman and Lambert (2000)
	Kibale NP, UGA	–	–	100	Oates (1977a, b)
	Entebbe, UGA	–	–	63	Grimes (2000)
	Budongo Forest, UGA	–	–	49	Suzuki (1979)
	Budongo Forest, UGA	428	39.3	–	Plumptre and Reynolds (1994)
	Budongo Forest, UGA	793	15.0	56	Hobaiter et al. (2017)
	lturi, COD	–	–	17	Bocian (1997)
	Dja Reserve, CMR	526	–	4.9	Poulsen et al. (2001)
*C. angolensis*	Ituri Forest, COD	–	1.2	7.7	Thomas (1991)
*C. a. palliatus*	Shimba Hills Nat. Reserve, GHA	–	2.9	15.3	Anderson et al. (2007)
	Okapi Faunal Reserve, COD	–	1.2	16.7	Bocian (1997)
*C. satanas anthracinus*	Lope Reserve, GAB	5360	0.75	11	Brugière (1998)
	Forêt des Abeilles, Makandé, GAB	–	–	7	Brugière et al. (2002)
	Taï National Park, CIV	–	2.8	47	Korstjens (2001)
*C. vellerosus*	Boabeng-Fiema, GHA	–	15	–	Wong and Sicotte (2006)

CMR: Cameroon; CIV: Côte d'Ivoire; COD: Democratic Republic of Congo; ETH: Ethiopia; GAB: Gabon; GHA: Ghana; KEN: Kenya; TZA: Tanzania; UGA: Uganda.

Generally, guerezas are ecologically relatively flexible, and they can survive even in small forest fragments (e.g., just a few trees surrounding a church in Ethiopia) (Dunbar and Dunbar, 1974; Fashing et al., 2019). They can also subsist in parks or can be found in tiny forest remnants in towns and are, in general, tolerant of the presence of humans (Yalden et al., 1977). Given the density of DMGs within the four study forests, conservation concern seems not to be a low population density per se but might be more the small range and the low number of suitable forests within the range of DMGs (Kufa et al., 2022). Habitat suitability models revealed that only 1336 km ${}^{2}$ (1.8 %) of the 75 307 km ${}^{2}$ study area consist of a highly suitable habitat for DMGs under current climate conditions (Kufa et al., 2022). Given that the species occurs mainly at higher altitudes, climate change can have additional negative effects on the habitat of DMGs, especially if the vegetation belts are “pushed” uphill, similar to what is expected for other high-altitude species in Ethiopia (Ahmed et al., 2023).

Because of their assumed limited geographic distribution (Zinner et al., 2019; Kufa et al., 2022) and therefore an assumed relatively small population size, DMG is most likely facing a higher risk of extinction than *C. g. guereza*. Also, the remnant forests where DMGs are found are isolated, making genetic exchange among the local populations of DMG difficult if not impossible. This can lead to an increase in inbreeding and a loss of genetic diversity. We therefore suggest a population genetic study to assess the genetic status of DMG. A comprehensive survey to collect samples for genetic analysis (noninvasive sampling, e.g., fecal material) could be the next step. We further suggest determining the geographic distribution of *C. g. gallarum*, because several forests that constitute a suitable habitat for DMG are unexplored, e.g., in the Arba-Gugu Mountains. It would also be important to collect data along the common distribution border between DMGs and other guereza taxa and to check for possible sympatry and hybridization. Finally, we recommend that conservation management programs focus on reconnecting forest fragments to re-establish dispersal routes among currently isolated local populations of DMGs. However, this should be accompanied by a public awareness campaign and discussions with the stakeholders involved.

## Supplement

10.5194/pb-10-13-2023-supplement The supplement related to this article is available online at: https://doi.org/10.5194/pb-10-13-2023-supplement.

## Data Availability

All raw data can be provided by the corresponding author upon reasonable request.
