# Supplementary material for: Djaffa Mountains guereza (Colobus guereza gallarum) abundance in forests of the Ahmar Mountains, Ethiopia
Source: Primate Biol. 2023 Oct 20;10(2):13–23. doi: 10.5194/pb-10-13-2023 (PMC10654609; doi:10.5194/pb-10-13-2023)
Supplement: The supplement related to this article is available online at: https://doi.org/10.5194/pb-10-13-2023-supplement. [file pb-10-13-supplement.pdf]

Supplement of Primate Biol., 10, 13–23, 2023
https://doi.org/10.5194/pb-10-13-2023-supplement
© Author(s) 2023. CC BY 4.0 License.

Supplement of

Djaffa Mountains guereza (*Colobus guereza gallarum*) abundance in forests of the Ahmar Mountains, Ethiopia

Chala Adugna Kufa et al.

Correspondence to: Chala Adugna Kufa (kchalaadugna@gmail.com)

The copyright of individual parts of the supplement might differ from the article licence.

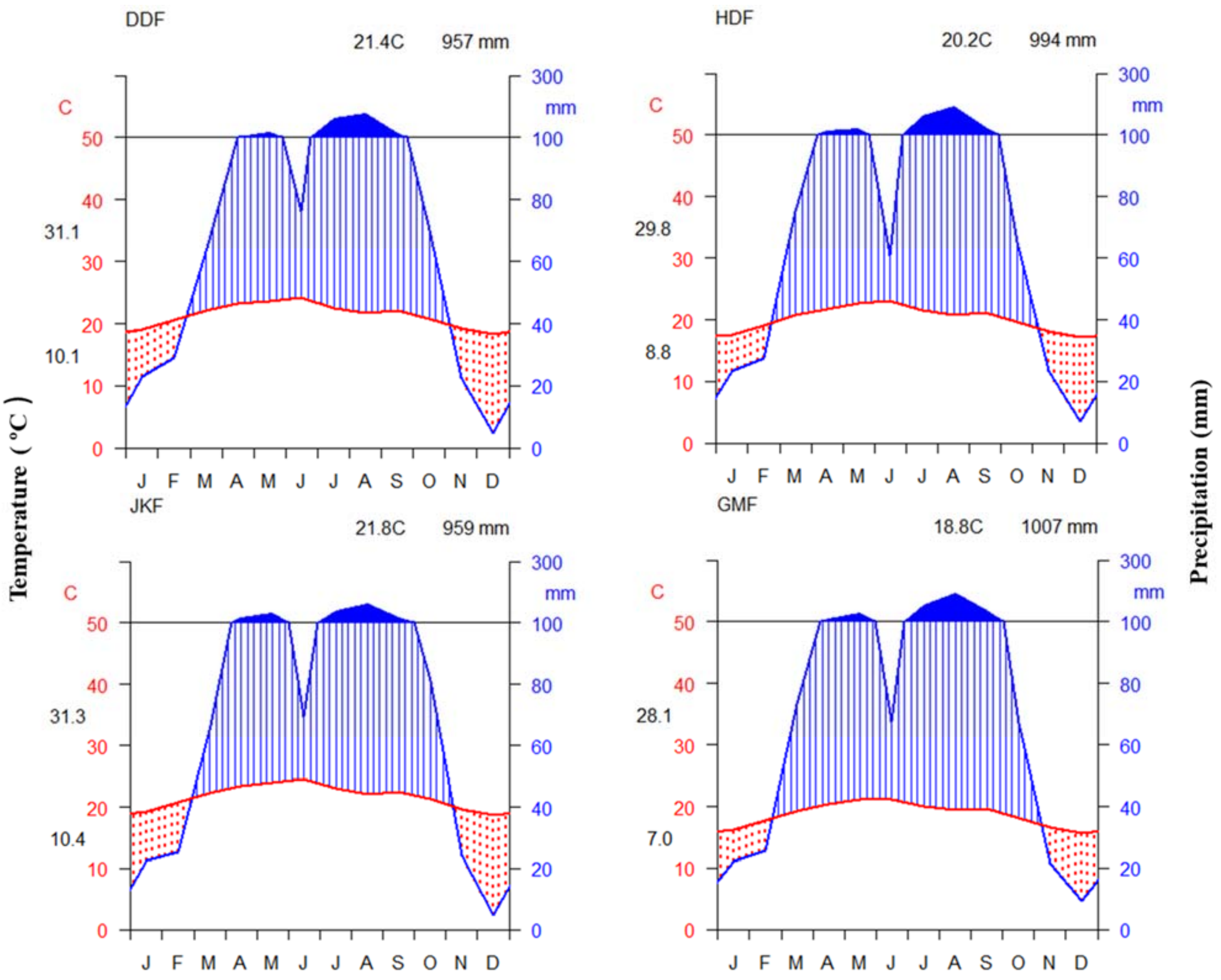

Fig. S1 Climate data of the four study sites, DDF, HDF, GMF, JKF in the Ahmar Mountains, Ethiopia, from 1989-2019. These climate diagrams include monthly mean temperature and precipitation. We downloaded the climate data from the Centre for Environmental Data Analysis (CEDA) datasets of the Climate Research Unit (University of East Anglia Climatic Research Unit; Harris et al., 2020). The black numbers on top of each diagram indicate annual mean temperatures and annual rainfall. The black numbers at the y-axis indicate the minimum and maximum temperatures (°C) of the respective coldest and warmest month.

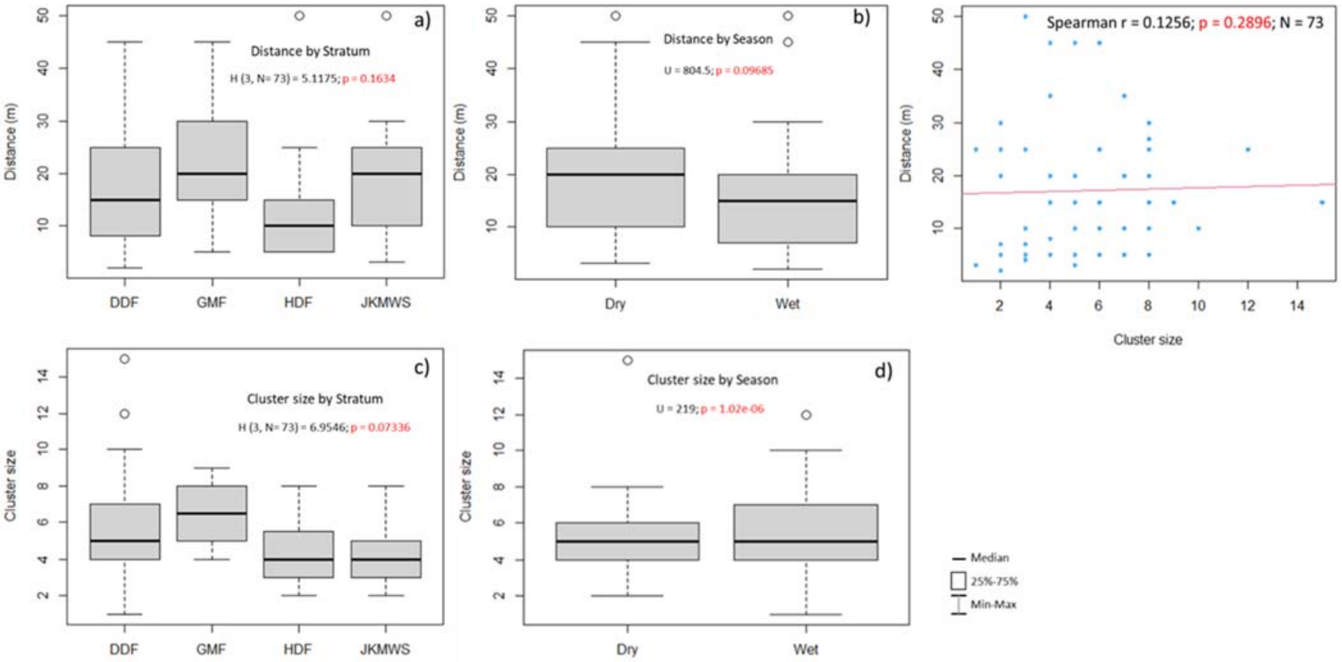

Fig. S2. Comparison of perpendicular distances (a and b) and cluster sizes (c and d) among the four forests and the two seasons and the correlation between cluster size and perpendicular distance (e).

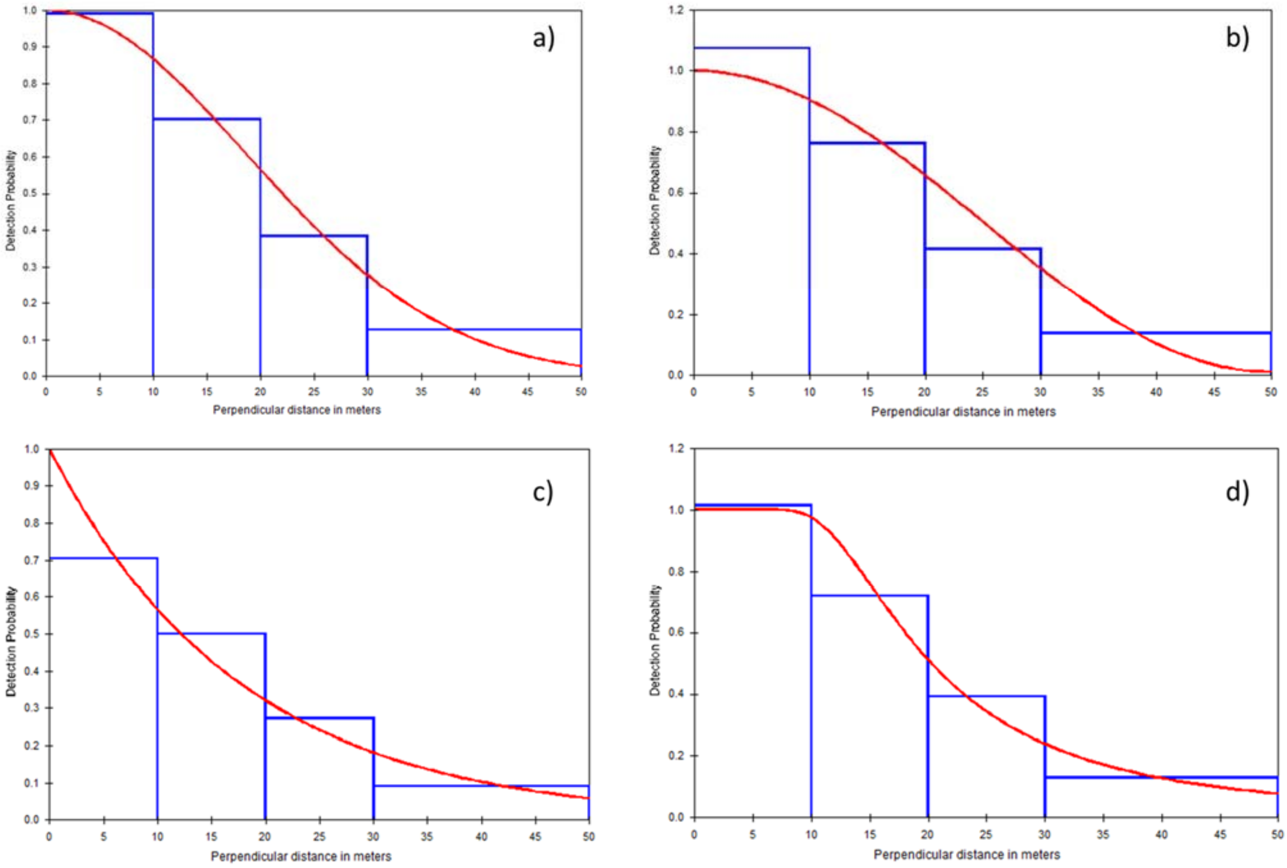

Fig. S3 The detection function models of Djaffa Mountains guereza fitted using different key functions for all data combined encountered during the field surveys across the forest fragments in the Ahmar Mountains, Eastern Ethiopia. (a) half normal, (b) uniform, (c) negative exponential, (d) hazard rate.

Table S1 Chi-square goodness of fit test for the four models (1-4) fitted to the perpendicular distance of Djaffa Mountain guereza survey data with right-truncation value = 50 m.

model	cut points	observed value	expected value	chi-square
1	0.0 – 10.0	31	29.89	0.041
2	10.0 – 20.0	22	22.64	0.018
3	20.0 – 30.0	12	12.98	0.074
4	30.0 – 50.0	8	7.49	0.035

Total Chi-square value = 0.1682, df = 2.00, P = 0.91932
